# Demand-side determinants of rising hospital admissions in Germany: the role of ageing

**DOI:** 10.1007/s10198-019-01033-6

**Published:** 2019-02-09

**Authors:** Jonas Krämer, Jonas Schreyögg

**Affiliations:** Hamburg Center for Health Economics, Universität Hamburg, 20354 Hamburg, Germany

**Keywords:** Hospital, Population age structure, Time to death, Morbidity, Inpatient admissions, I11, J11

## Abstract

In this study, we investigated the relationship between changes in demand-side determinants and changes in hospital admissions. We used longitudinal market-wide data, including a novel detailed measure of population morbidity. To assess the effect of ageing, we interacted age with shifts in the population structure for both the surviving population and the population in their last year of life. We used fixed effects models and addressed the endogeneity of morbidity with instrumental variables. We found that changes in morbidity had the largest impact on changes in hospital admissions. Changes in the size of the surviving population had the second largest impact, which differed substantially across the age spectrum. There was a large response in admissions to changes in the size of the population aged 60–79 years. The end-of-life effect had the smallest impact and began to play a greater role only in the population aged 80 years and older. In many studies, end of life presumably approximates high morbidity. Our results demonstrated robustness in several tests. We performed estimations in separate major diagnostic categories and included changes in personal preferences. We argue that the determinants included in our estimations capture the vast majority of change on the demand side. Taken together, our findings provide evidence that these determinants explain one-fifth of changes in hospital admissions.

## Introduction

A frequent assumption in health policy circles and health services research is that demand for health care services will increase as the population in most developed countries ages, leading to pressure to increase public spending on these services in the future. Approximately, one-third of all health care expenditure (HCE) in OECD countries is in inpatient services [1]. In Germany, inpatient admissions increased steadily from 16.5 million in 2005 to 19.2 million in 2015, representing by far the largest percentage increase in hospital admissions among OECD members, even though the size of the population has remained the same [2]. The extent to which this tremendous growth has been driven by changes in population demand or hospital supply, however, remains largely unclear.

The majority of recent studies have concluded that the role of demand-side or patient factors is limited and that the supply side plays a more important role in driving variation and growth of health care utilization and expenditure in advanced economies [3–5]. Germany introduced a system of diagnosis-related groups (DRGs) to set prices for most inpatient services in 2005. Within DRG-based payment systems, hospitals may change the number of admissions at the extensive margin in response to price changes [6]. Indeed, they have a major incentive to increase the number of admitted patients, because hospital revenue in such systems is determined by multiplying the activity of each DRG by the fixed payment per DRG [7].

Various demand-side determinants have been studied at the micro- and macro-levels. Most studies have attempted to explain inpatient health care utilization in terms of expenditure. For instance, de Meijer et al. [8] reviewed factors associated with an ageing population. The identified demand-side factors should also be applicable in our study to explain changes in inpatient admissions. Because national or regional budgets for inpatient care are often set by health insurers or governments and they determine which technologies are supplied to patients [9], the relationship between demand and hospital admissions should actually be more direct than the relationship between demand and HCE. Germany has almost complete health insurance coverage of its population; therefore, income and demand response to prices have little effect. Co-payments are limited to a few items in German public insurance [10]^1^.

One major stream of literature is centred around the “red herring” hypothesis. In a seminal paper, Zweifel et al. [11] concluded that the remaining lifetime, or time to death (TTD), and not the calendar age per se mattered more when explaining health care utilization. While numerous studies have addressed the “red herring” claim, a shortcoming of many of these is their reliance on cross-sectional data. However, several longitudinal studies have evaluated the influence of TTD on hospital expenditure as well. For example, Seshamani and Gray [12] used a random effects panel model and found that proximity to death was strongly associated with hospital costs even 15 years before death. Felder et al. [13] investigated the possible endogeneity of TTD, and Wong et al. [14] analysed disease-specific hospital expenditure and found that age was a minor determinant of HCE compared to the TTD. However, the finding that TTD explains health care utilization better than age is not surprising considering that both variables are used as approximations of health status, which is the real driver of individual health care demand but is more difficult to measure. Recent studies have confirmed that TTD loses its explanatory power when analyses control for morbidity [9].

Thus, another stream of literature has evaluated the effects of age on HCE while controlling for health status [15]. Lubitz et al. [16] related hospital costs to the current health status of 70-year-old patients. The authors found that better health did not necessarily lead to lower health care costs. Dormont et al. [17] used chronic illnesses and disability indicators as a measure of morbidity and found that HCE no longer increased with age when controlling for morbidity. To investigate the longitudinal relationship between health and the costs of health care use, Wouterse et al. [18] combined survey data with information from the Dutch national hospital register. These authors included mortality and different indicators of morbidity to measure initial health status and found that for age groups up to 70 years, pre-existing poor health led to higher costs of hospital use, which persisted over an 8-year period.

Studies are largely motivated by explaining the growth of HCEs. Very few studies, however, have explicitly investigated how changes in HCE are related to changes in demand-side determinants over time. Examples include the studies by Van Baal and Wong [9] and de Meijer et al. [19], who decompose changes in the distribution of HCE between the periods 1998 and 2004 using Dutch data on HCE linked to hospital discharge and mortality registers. They found that growth in hospital expenditure was greatest at the middle of the distribution and could be explained by changes in the observed determinants of expenditure. However, they found that population characteristics accounted for only approximately one-fifth of HCE growth.

Analyses investigating the “red herring” claim or demand-side determinants usually prefer to use individual-level samples and two-part models. Other studies argue that the most profound implications have been drawn using macro-level data [9]. The use of samples from individual hospitals or statutory health insurers, which represent only parts of the market, is problematic. Finkelstein [20] demonstrated how conclusions from market-wide analyses might deviate from partial market analyses [20]. The use of macro-level data instead of individual-level samples has the advantage of providing findings that are representative of the population as a whole and allows more generalizable implications to be drawn [21, 22]. For instance, van Baal and Wong [9] used a macro-level approach to forecast HCE based on differences in the TTD using aggregate-level panel data from the Netherlands (i.e., age- and gender-specific per capita hospital expenditures and mortality rates).

We contribute to the literature using an extensive market-wide dataset. Our data allowed us to observe the entire German population at a very detailed level and to relate age–gender–county-specific changes over time in demand-side determinants to age–gender–county-specific changes in hospital admissions. We aggregated 80 million people into almost 80,000 groups that we observed over a 4-year period. Moreover, our data covered all hospitals and therefore market-wide inpatient admissions for both publicly and privately insured patients regardless of age or income were also included. The aim of our study was to explain annual changes in hospital admissions using the annual changes in all major demand-side determinants identified in a previous research (i.e., morbidity, the “red herring” claim or end-of-life effect, changes in population structure and the effect of ageing).

In addition to using an extensive market-wide dataset, our study has three important advantages relative to the previous literature. First, we support the notion that TTD and age are only crude estimators of high hospital utilization, because these measures approximate health status, which is the variable that really matters [14]. A unique feature of our study is its novel, detailed measure of population health status, in terms of morbidity, over time. Second, as discussed by Finkelstein et al. [4], evidence that morbidity drives health care utilization has been inconclusive, because the prevalence of a diagnosis is itself endogenous. We aimed to overcome the endogeneity problem between changes in morbidity and changes in hospital admissions using instrumental variables. Third, as emphasized by de Meijer et al. [8], ageing most likely influences growth in health expenditure indirectly by interacting with other factors. Instead of estimating associations between health care utilization and age alone, we interacted age with changes in the surviving population and in the end-of-life population (i.e., people in their last year of life). This approach provides an explicit measure of demographic change and allowed us to determine the importance of the surviving population as a predictor compared to the end-of-life effect over the entire age spectrum. Finally, we performed a number of robustness checks that also included further data sources. For instance, we included changes in personal preferences and provided separate analyses for hospital admissions in all major diagnostic categories (MDCs).

## Data

We used a variety of data sources to construct an age–gender–county-specific dataset for annual changes that occurred during the period from 2008 to 2011. All datasets covered the full German population with the exception of that from the German Socio-Economic Panel (SOEP). Table 1 gives descriptive statistics for all variables and years, as well as the changes that occurred from 2008 to 2011. We obtained data on hospital admissions through administrative reimbursement data covering all inpatient admissions in Germany from 2008 to 2011 (both cumulatively and separately) for all MDCs. Lastly, we collected the population data and numbers of people in their last year of life for all age–gender combinations in each county from the German Federal Statistical Office.

**Table 1:** Descriptive statistics

Year	2008	2009	2010	2011	∆2008–2011 (%)
Inpatient admissions (sum)	15,589,612	15,856,047	16,091,887	16,379,713	5.07
Morbidity (mean)	0.9714	0.9670	0.9618	0.9592	− 1.26
Age (mean)	42.9386	43.1917	43.4347	43.6573	1.67
Unemployment (mean)	0.0802	0.0678	0.0591	0.0619	− 1.83
Risk behaviour (mean)	4.4906	3.7900	4.3691	4.6063	2.58
Population (sum)	81,176,785	80,986,130	80,915,133	81,022,618	− 0.19
Surviving population (sum)	80,384,835	80,185,541	80,111,233	80,224,485	− 0.20
End-of-life population (sum)	791,950	800,589	803,900	798,133	0.78

To measure changes in population morbidity, we relied upon the Risk Structure Compensation Scheme (RSA) risk factor used to allocate funds between Germany’s statutory health insurers. The risk factor reflects the morbidity of all publicly insured patients measured in terms of inpatient and outpatient diagnoses for 80 important illnesses. Outpatient diagnoses must be claimed in at least two quarters during the same year and validated by defined pharmaceutical prescriptions. The risk factor is attractive because it measures health status as determined by doctors in their claims. It is comprehensive and can be assumed to be highly accurate [21]. A detailed description can be found in Göpffarth et al. [21]. For the purpose of our analysis, we used a recalculated version of the risk factor obtained from the National Association of Statutory Health Insurance Physicians, which we call the morbidity index. While Göpffarth et al. [21] used the risk factor at the county level in 2011, we calculated our morbidity index at the level of age, gender and county from 2008 to 2011 relative to the base year. Our index shows how morbidity in these groups deviates from the average morbidity of all publicly insured patients. We limited it to information from outpatient care to mitigate endogeneity with inpatient admissions.

We obtained data on unemployment rates from the German Federal Statistical Office. To measure changes in personal preferences, we followed the approach of Göpffarth et al. [21] and used risk behaviour from the SOEP. Data on people older than 95 years were unavailable, and we excluded newborns from the analysis. To best describe population demand, we linked hospital admissions to all other data sources in the patient’s county. To observe comparable changes over time, we related all of the data sources to the 412 German counties in the 2010 version.

## Methods

### Model specification

The general objective of our model was to obtain unbiased estimates of all major demand-side determinants of changes in hospital admissions. To link all of the data sources, we aggregated our variables into age–gender–county groups in a synthetic panel similar to that described by Deaton [23]. We included 95 separate age groups for men and women in 412 counties for a total of 74,855 observations from 2008 to 2011.^2^ Each observation represented approximately 1000 people on average. We took the log and first difference of all variables to obtain the percentage change at level $i$ (age-gender-county) from year $t - 1$ to year $t$. We denoted $\Delta {A_{it}}$, which is the percentage change in admissions, as our dependent variable^3^ and $\Delta {M_{it}}$ as the percentage change in the morbidity index. We expected an increase in inpatient admissions if population morbidity worsened as measured using outpatient claims. To test the “red herring” hypothesis, we split the population into the surviving population (surviving in a given year) and end-of-life population (deceased within a given year)^4^. $\Delta {P_{it}}$ was the change in the population that was not in the last year of life, and $\Delta {D_{it}}$ the change in the end-of-life population^5^. Clearly, we expected admissions to increase if the size of the (surviving) population increased, and we expected an increase in admissions due to the end-of-life effect as measured by an increase in the size of the population in the last year of life.

We used a fixed effects model to control for potential omitted variable bias. Annual fixed effects allowed us to separate demand-side responses in admissions from time trends associated with omitted factors, such as changes in hospital supply or technological progress. We also controlled for unobserved heterogeneity among each combination of age, gender and county. Our basic estimating equation was:

1 $$\Delta {A_{it}}={\beta _1}\Delta {M_{it}}+{\beta _2}\Delta {D_{it}}+{\beta _3}\Delta {P_{it}}+{c_j}+{u_i}+{\mu _t}+{\vartheta _{it}},$$

where ${u_i}$ are fixed effects (age–gender–county), and ${c_j}$ are gender cohort dummies from 1915 to 2010^6^. The marginal effects of changes in morbidity, surviving and end-of-life population sizes on changes in admissions are revealed in ${\beta _{1 - 3}}$. There are $t - 1~$ year-specific dummy variables (${\mu _t})$. We frequency weighted all observations by the underlying population size, and the standard errors were clustered at the age–gender–county levels.

Due to the richness of our data, we were able to depict the effect of ageing or the demographic changes at a very detailed level. Shifts in the population structure for the short period from 2008 to 2011 were predominantly a result of different birth rates rather than longer life expectancy. Nevertheless, we observed shifts in the population structure and the corresponding age-specific changes in hospital admissions in over 400 counties. Therefore, an alternative specification was to complement Eq. (1) by interacting changes in both the surviving and end-of-life populations to allow for the possibility that age influenced the way that changes in the population affected changes in hospital admissions. We did not assume age to be linear or another functional form, and we used dummy coding to allow for maximum flexibility. The interaction between changes in the size of the surviving population ($\Delta {P_{it}})$ and 5-year age groups (*a*) yielded an explicit measure of the consequences of demographic change (i.e., does an increase in people in older age groups lead to a greater increase in admissions than an increase in people in younger age groups?). The marginal effects of changes in population size in different age groups on changes in hospital admissions are represented by $\mathop \sum \nolimits_{a} {\beta _{3a}}$. Additionally, the literature suggests that the end-of-life effect may be different for different age groups [9, 22]. Therefore, we allowed the interaction between $\Delta {D_{it}}~$. and the 5-year age groups (*a*), which gave the following estimating equation:

2 $$\Delta {A_{it}}={\beta _1}\Delta {M_{it}}+\mathop \sum \limits_{a} {\beta _{2a}}\Delta {D_{it}}+\mathop \sum \limits_{a} {\beta _{3a}}\Delta {P_{it}}+{c_j}+{u_i}~+{\mu _t}+{\vartheta _{it}}.$$

One important benefit of our study is our detailed measure of change in population morbidity. However, this change is probably endogenous with the change in hospital admissions, since hospitals may be able to help keep morbidity stable or even to reduce morbidity. Therefore, we speculated that ${\beta _1}$ was underestimated. Moreover, measurement errors in the morbidity score might be expected in the event of regional differences in coding quality [24]. We took both possibilities into account by estimating our fixed effects (FE) model with instrumental variables in two-stage least squares (2SLS). We used the change in the unemployment rate as an instrument for the change in morbidity. The literature suggests that the unemployment rate has a positive effect on (i.e., worsens) morbidity, albeit with a lag of 1–5, or 2–3, years (i.e., deterioration in health status is observed 2–3 years after an increase in unemployment) [25, 26].

Accordingly, we found that changes in unemployment had a positive significant influence on morbidity at all lags from 1 to 3 years in our data. The effect size for the 1-year and 3-year lags was rather small, but we found the largest effect size for a lag of 2 years. We did not expect a direct link from changes in the lagged unemployment rate to changes in hospital admissions other than through morbidity. In our data, the unemployment rate had no significant influence on the end-of-life effect for any of the lags. Therefore, we chose the change in the 2-year lagged unemployment rate as an instrument for change in morbidity, as we felt this was best grounded in theory and empirically supported. Felder et al. [13] have discussed the potential endogeneity of TTD and health care utilization and considered this to be a minor problem. Unfortunately, we lacked valid instruments to address this. We assumed, however, that this endogeneity problem was less severe than those associated with morbidity. Germany has a rich supply of hospitals, waiting times hardly exist for inpatient care, and patients with life-threatening conditions are rarely denied admission. We estimated all of the models shown in Table 2 using the full sample. For the robustness checks presented in the next section, we used different, reduced samples.

**Table 2:** Determinants of changes in hospital admissions

Specification	FE	FE/2SLS	FE	FE/2SLS
Column	1	2	3	4
∆Morbidity	0.137*** (9.85)	0.768** (2.92)	0.137*** (9.92)	0.747** (2.83)
∆End-of-life population	0.0175*** (23.61)	0.0172*** (22.91)	–	–
∆Surviving population	0.174*** (25.07)	0.174*** (24.85)	–	–
∆Surviving population in age group				
1–4			0.2656*** (4.76)	0.2755*** (4.93)
5–9			0.2864*** (5.48)	0.2866*** (5.48)
10–14			0.1985*** (3.99)	0.1898*** (3.80)
15–19			0.3225*** (10.35)	0.3327*** (10.51)
20–24			0.2213*** (7.39)	0.216*** (7.16)
25–29			0.2133*** (6.01)	0.219*** (6.14)
30–34			0.1804*** (5.32)	0.176*** (5.15)
35–39			0.1511*** (4.35)	0.1454*** (4.16)
40–44			0.1579*** (4.43)	0.1672*** (4.63)
45–49			0.1468*** (4.15)	0.1461*** (4.14)
50–54			0.1413*** (4.29)	0.1406*** (4.24)
55–59			0.1862*** (6.24)	0.1719*** (5.60)
60–64			0.2944*** (18.02)	0.2967*** (18.05)
65–69			0.2553*** (14.74)	0.242*** (13.15)
70–74			0.1637*** (7.50)	0.1917*** (7.54)
75–79			0.22*** (10.92)	0.2067*** (9.66)
80–84			0.1522*** (8.95)	0.1591*** (8.94)
85–89			0.1084*** (7.31)	0.1008*** (6.48)
90–95			0.0843*** (5.64)	0.0836*** (5.55)
∆End-of-life population in age group				
1–4			0.0153 (1.57)	0.0149 (1.48)
5–9			0.0379 (1.52)	0.0359 (1.44)
10–14			0.0168 (0.98)	0.017 (0.99)
15–19			− 0.0034 (− 0.53)	− 0.0032 (− 0.50)
20–24			0.0029 (0.91)	0.0032 (1.02)
25–29			0.011** (2.69)	0.01* (2.47)
30–34			0.0029 (0.79)	0.0032 (0.88)
35–39			0.0038 (1.43)	0.0036 (1.36)
40–44			0.0095*** (5.21)	0.0092*** (5.07)
45–49			0.0134*** (8.21)	0.0132*** (8.03)
50–54			0.0177*** (9.80)	0.0173*** (9.46)
55–59			0.0245*** (12.19)	0.0237*** (11.58)
60–64			0.0354*** (14.90)	0.0352*** (14.64)
65–69			0.046*** (16.07)	0.0458*** (15.67)
70–74			0.0619*** (16.60)	0.0624*** (16.35)
75–79			0.1092*** (23.37)	0.109*** (22.63)
80–84			0.1437*** (24.08)	0.145*** (23.53)
85–89			0.2015*** (27.34)	0.1974*** (25.44)
90–95			0.2497*** (22.17)	0.2533*** (22.21)
${\alpha _i}$	Included	Included	Included	Included
${c_j}$	Included	Included	Included	Included
${\mu _t}=2009$	− 0.00196* (− 2.23)	− 0.00333** (− 3.03)	− 0.00177* (− 2.00)	− 0.00315** (− 2.81)
${\mu _t}=2010$	− 0.00613*** (− 6.28)	− 0.00653*** (− 6.49)	− 0.00607*** (− 6.22)	− 0.00651*** (− 6.45)
${\mu _t}=2011$	Base	Base	Base	Base
*N*	74,855	74,855	74,855	74,855

Numbers in brackets represent *t* values

Two-tailed significance levels: *p < 0.05; **p < 0.01; ***p < 0.001

### Robustness checks

We performed robustness checks to examine whether our estimates suffered from omitted variable bias. First, we tested whether our results critically depended on including changes in the size of the population more than 1 year from death. Evidence suggests that the strongest effect occurs in the last year of life, although there is also evidence for positive effects in the 2–15 years before death (e.g., [12]). Using data from the deceased from future years, we could express the change in the number of people in their second-to-last year of life in a given year and the corresponding survivors. While we would have preferred to have included the parts of the population more distant from death, including the second-to-last year of life unfortunately restricted our sample to a minimum of two changes: from 2008 to 2009 and from 2009 to 2010. We estimated models (1) and (2) with that subsample. Second, we tested whether omitting changes in personal preferences as a demand-side factor that can influence individual health behaviours may have biased the results of our main estimation [8]. We measured personal preferences using changes in risk aversion captured in the SOEP data and included this as a variable in our robustness test. Because these data covered observations only from people aged 18 years and older, we excluded 15% of our observations and estimated model (2) using this subsample. For our third robustness test, we investigated how the demand-side determinants differed among the different diagnostic categories. We regressed changes in admissions for separate MDCs on the changes in the demand-side determinants. Wong et al. [14] showed highly heterogeneous results for different categories of illness treated in the hospital. We used model (1) and excluded MDCs 14 and 15 (“Pregnancy, Childbirth and Puerperium” and “Newborns and Other Neonates (Perinatal Period)”), because these MDCs applied only to small fractions of the population. We also excluded MDCs 16 and 17 from our analysis (“Transplants” and “Extensive Procedures Unrelated to Principal Diagnosis”), since these categories did not exist in Germany.

## Results

The regression results are shown in Table 2. Column 1 presents the FE regression results for model (1), and column 2 presents the corresponding 2SLS results. Columns 3 and 4 show the FE and 2SLS regression results for model (2), including the age group-specific interactions. Generally, our estimates suggest that changes in morbidity had the largest impact on changes in hospital admissions. The effects of changes in the population differed substantially over the age spectrum. Regarding the surviving population, the largest percentage increase in admissions was significantly associated with an increase in the size of the population of children, especially teenagers, and to an increase in the size of the population in the 60–79 year age group. An increase in the size of the end-of-life population generally had the smallest impact, albeit one that increased with age: this effect began to play a role in people 40 years of age, but only had a larger impact than the surviving population in people 80 years of age or older.

The FE estimation for morbidity can be found in Table 2 in columns 1 and 3. The 2SLS results using changes in the unemployment rate as an instrument for changes in morbidity are shown in Table 2 in columns 2 and 4 (main results) and Appendix Table 4 in columns 1 and 2 (first-stage results). Tests for the validity of the instrument (over-identifying restriction tests) were not applicable in our case because we used a single instrument. However, an empirical check suggested that we can be confident in the results: the instrument was sufficiently strong in both specifications, with an increase in lagged unemployment leading to a highly significant increase in morbidity. To confirm the strength of our instrument, we performed the Kleibergen–Paap test for weak instruments for both specifications [27]. For a single endogenous variable that is exactly identified, Stock and Yogo report that the critical value of the *F* test is 16.38. The test clearly rejected the null hypothesis that the instruments were weak (*F* test, 501.86 and 493.46). The strength of our instruments in the first-stage regressions was such that the maximum relative bias was less than 10% [28]. As we expected, the coefficient representing an endogenous morbidity effect that increased from 0.14 to 0.75, indicating that a 10% increase in morbidity on average leads to a 7.5% increase in admissions (ceteris paribus, column 4). We found that coefficients and standard errors remained consistent and robust for all of the different specifications (Table 2, columns 2 and 4 and Table 3, columns 3–5).

**Table 3:** Robustness checks for determinants of changes in hospital admissions

Specification	FE	FE	FE/2SLS	FE/2SLS	FE/2SLS
Column	1	2	3	4	5
∆Morbidity	0.211*** (8.22)	0.212*** (8.26)	0.627** (3.15)	0.626** (3.13)	0.649** (3.18)
∆End-of-life population	0.0201*** (15.19)	0.0179*** (18.09)	0.0172*** (22.98)	–	0.0172*** (22.96)
∆Surviving population	0.123*** (13.44)	0.122*** (13.39)	0.169*** (24.10)	–	0.169*** (24.07)
∆Second-to-last year of life population	0.00326* (2.45)				
∆Preferences			− 0.00802* (− 2.05)	− 0.00856* (− 2.20)	
∆Surviving population in age group					
18–19				0.3187*** (10.12)	
20–24				0.2187*** (7.28)	
25–29				0.2154*** (6.04)	
30–34				0.1749*** (5.11)	
35–39				0.1701*** (4.88)	
40–44				0.1499*** (4.21)	
45–49				0.1468*** (4.15)	
50–54				0.1416*** (4.30)	
55–59				0.1816*** (6.03)	
60–64				0.2956*** (18.07)	
65–69				0.2504*** (13.97)	
70–74				0.1715*** (7.52)	
75–79				0.2178*** (10.71)	
80–84				0.1541*** (9.00)	
85–89				0.1033*** (6.63)	
90–95				0.0884*** (5.28)	
∆End-of-life population in age group					
18–19				− 0.0028 (− 0.44)	
20–24				0.003 (0.93)	
25–29				0.0108** (2.64)	
30–34				0.003 (0.84)	
35–39				0.0036 (1.39)	
40–44				0.0093*** (5.14)	
45–49				0.0133*** (8.16)	
50–54				0.0176*** (9.66)	
55–59				0.0242*** (11.96)	
60–64				0.0354*** (14.86)	
65–69				0.0458*** (15.91)	
70–74				0.0619*** (16.52)	
75–79				0.1092*** (23.20)	
80–84				0.1441*** (23.97)	
85–89				0.1984*** (25.95)	
90–95				0.2487*** (19.28)	
${u_i}$	Included	Included	Included	Included	
${c_j}$	Included	Included	Included	Included	
${\mu _t}=2009$	0.00443*** (4.37)	0.00444*** (4.38)	− 0.00276* (− 2.28)	− 0.00124 (− 1.38)	− 0.00293* (− 2.39)
${\mu _t}=2010$	Base	Base	− 0.00384*** (− 3.61)	− 0.00440*** (− 4.43)	− 0.00397*** (− 3.75)
${\mu _t}=2011$	–	–	Base	Base	Base
*N*	74,142	74,142	63,621	63,621	63,621

Numbers in brackets represent *t* values

Two-tailed significance levels: *p < 0.05; **p < 0.01; ***p < 0.001

**Fig. 1:**
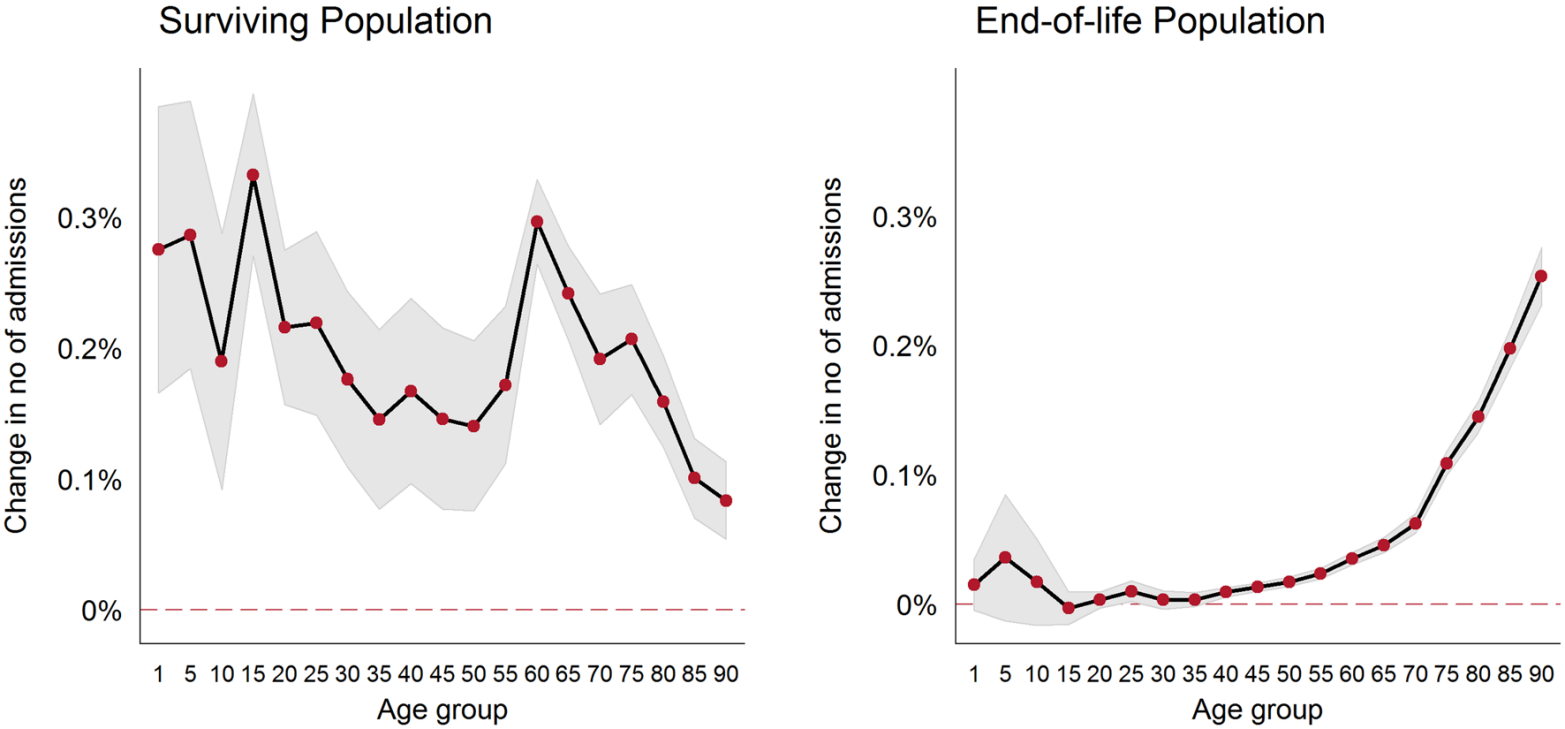
Age-specific effects of changes in the size of the surviving population and end-of-life population on changes in hospital admissions

Our estimates in columns 1 and 2 indicate that changes in the size of the surviving population overall led to tenfold larger changes in admissions compared with changes in the size of the end-of-life population. The similar results obtained for the age group interactions in columns 3 and 4 provide more insight into the effect sizes over the age spectrum. Figure 1 illustrates the marginal effects for the age group interactions from column 4 with their 95% confidence intervals. The left side shows the interaction of changes in the surviving population, and the right side shows the changes in the end-of-life population. For example, a 10% increase in the size of the population aged 75–79 and not in their last year of life led to a 2.1% increase in hospital admissions, whereas a 10% increase in the size of the population aged 75–79 who were in their last year of life led to a 1.1% increase in hospital admissions.

We found an age effect for the surviving population in terms of significant differences among the age groups but no linear or increasing age effect. Remarkable differences in age were found for teenagers (15–19 years) compared to the adjacent age groups. We also found a sharp increase in admissions among people aged 20–59 years to people aged 60 and 79 years. The effects of changes in the end-of-life population on admissions strongly and significantly increased with age as a consequence of the larger number of deceased persons among the older age groups. Although the uncertainty in the estimates for the surviving population was relatively high, we estimated the end-of-life effect precisely for all age groups above 40 years.

**Fig. 2:**
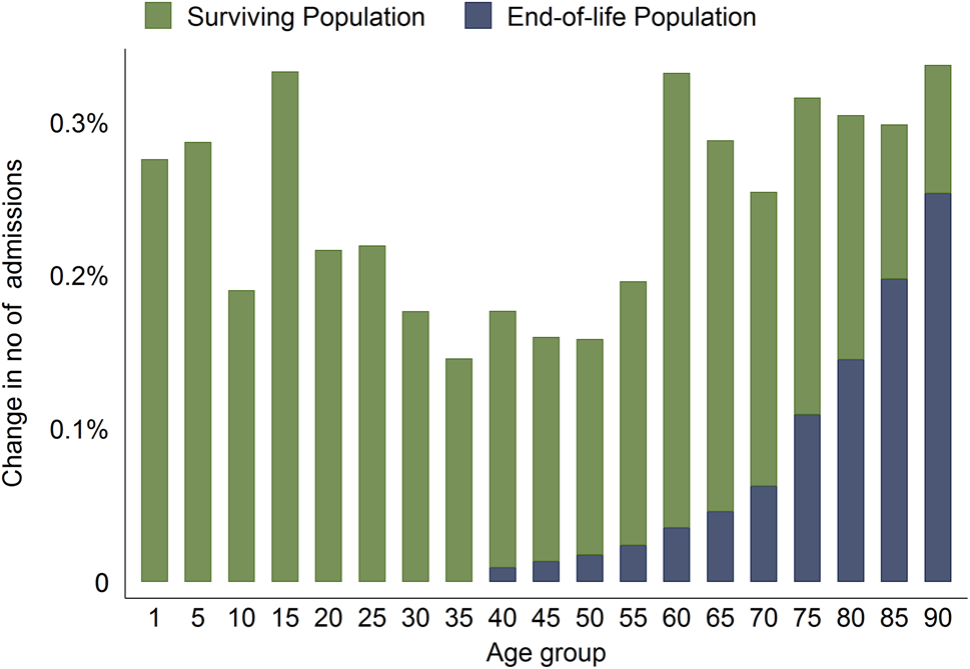
Composition of the population effects on changes in hospital admissions

Figure 2 illustrates the effects of both the surviving and the end-of-life populations. The effect of the end-of-life population was irrelevant below 40 years of age. In the group aged 60–64 years, the end-of-life effect accounted for approximately 10% of the cumulative population effect, whereas in the group aged 80–84 years it accounted for approximately 50% and in the oldest age group, aged 90–95 years, for 75% of the cumulative population effect. The combined population effect suggests a slightly U-shaped association between age and changes in admissions. We found the largest impact on admissions in the oldest groups, namely people between 60 and 95 years of age. Their average increase in admissions was 3% when both the surviving and the end-of-life populations increased by 10%. Among the younger age groups, aged 1–29 years, the average increase in admissions was 2.5%. The lowest impact was found for middle-aged persons, aged 30–59 years, who experienced an average increase in admissions of 1.7%.

**Fig. 3:**
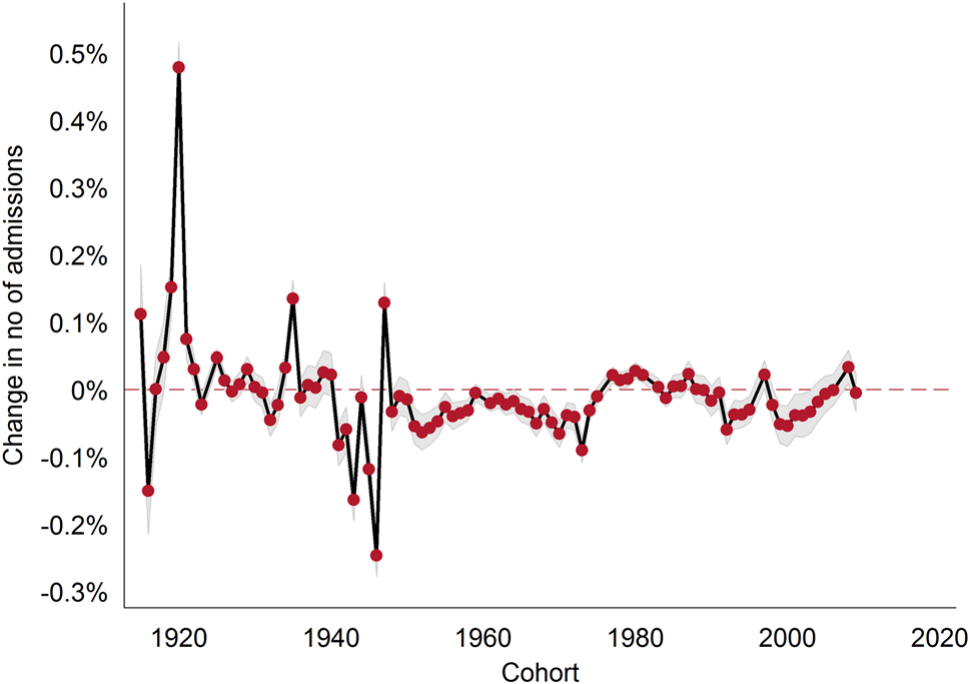
Cohort effects

Controlling for cohort effects is important. We found large effects for cohorts born during or directly after the First and Second World Wars (cohorts 1915–1920 and 1941–1947) (see Fig. 3), which is in line with the previous literature.

### Robustness checks

**Fig. 4:**
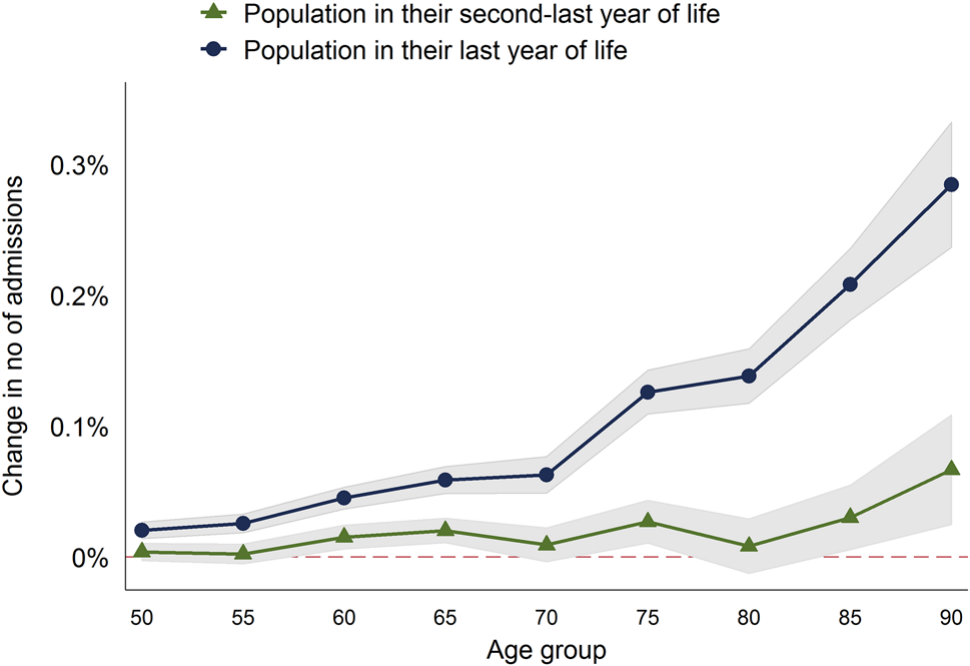
Age-specific effects of changes in the size of the populations in the last and second-last years of life on changes in hospital admissions

Table 3 shows the results obtained using the FE estimation and model (1) including (column 1) and not including the changes in the population in the second-to-last year of life (column 2). We found that the impact of the population in their second-to-last year of life was less than one-sixth of the impact of the population in their last year of life. This effect also had relatively weak significance. The other coefficients proved to be robust. Figure 4 illustrates the age-specific effects using model (2), which confirm our findings. Changes in the population in their second-to-last year of life had a minor impact on hospital admissions. Therefore, we concluded that the end-of-life effect was adequately depicted using the population in their last year of life.

As a second robustness check, columns 3 and 4 show the results when we included the changes in personal preferences, which may have influenced individual health behaviour in the model (1) and (2) 2SLS estimations. We found small significant negative coefficients, i.e., increasing risk aversion was associated with more hospital admissions. Personal preferences influence individual health behaviour and since we controlled for morbidity, this direction was plausible. Given the same state of health, risk-averse individuals demand more hospital services [21]. Without controlling for morbidity, one would theoretically expect riskier health behaviour to lead to higher morbidity and, therefore, to higher demand for hospital services. These individuals may depict the risk averse as having non-urgent admissions from the emergency department for further examinations (e.g., in case of back pain). However, the impact was small, and the other coefficients hardly changed when personal preferences were included (compare column 5). Additionally, our results proved to be robust when children were excluded from the analysis.

**Fig. 5:**
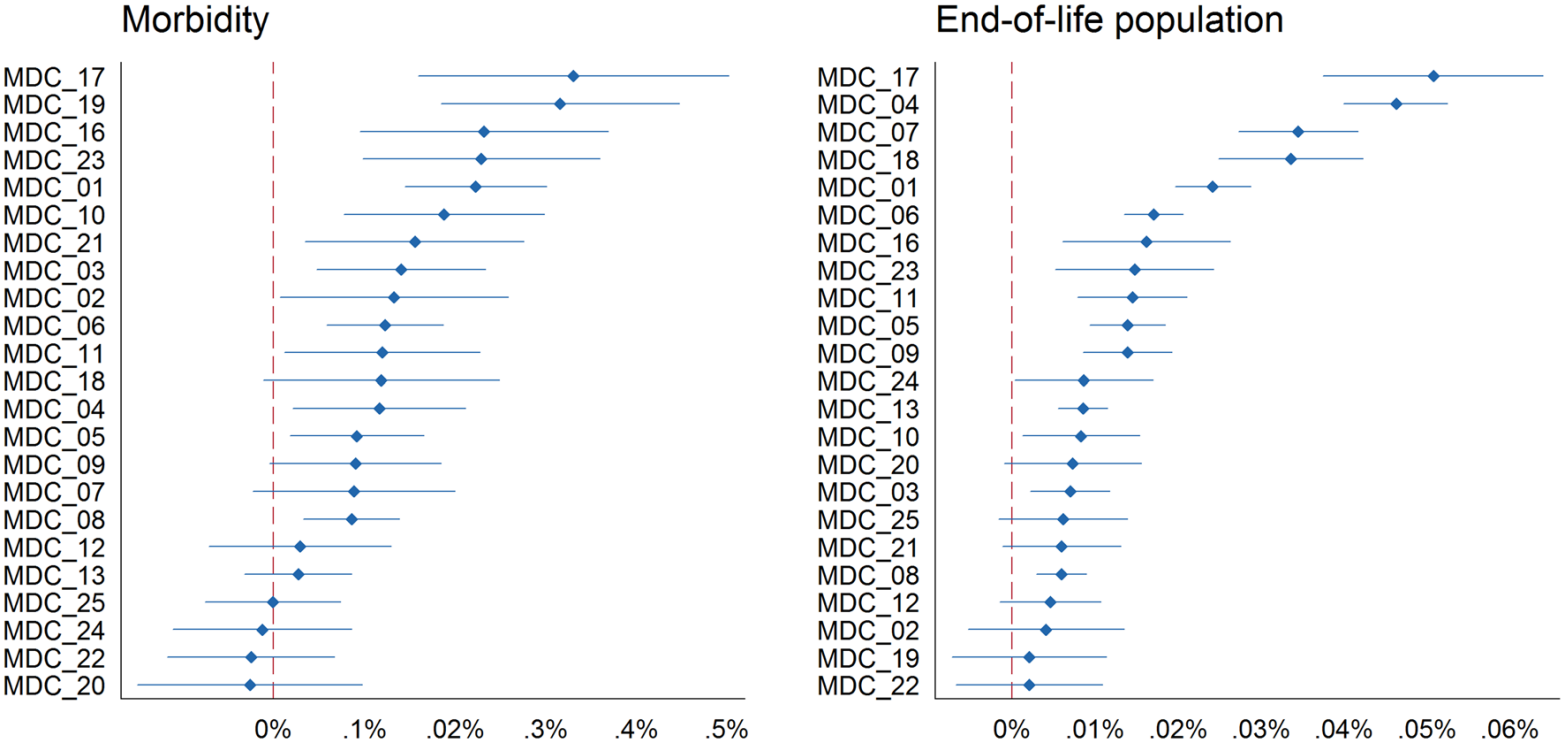
MDC-specific (MDC description: 01: Diseases and Disorders of the Nervous System; 02: Diseases and Disorders of the Eye; 03: Diseases and Disorders of the Ear, Nose, Mouth And Throat; 04: Diseases and Disorders of the Respiratory System; 05: Diseases and Disorders of the Circulatory System; 06: Diseases and Disorders of the Digestive System; 07: Diseases and Disorders of the Hepatobiliary System And Pancreas; 08: Diseases and Disorders of the Musculoskeletal System And Connective Tissue; 09: Diseases and Disorders of the Skin, Subcutaneous Tissue And Breast; 10: Diseases and Disorders of the Endocrine, Nutritional And Metabolic System; 11: Diseases and Disorders of the Kidney And Urinary Tract; 12: Diseases and Disorders of the Male Reproductive System; 13: Diseases and Disorders of the Female Reproductive System; 16: Diseases and Disorders of the Blood and Blood Forming Organs and Immunological Disorders; 17: Myeloproliferative DDs (Poorly Differentiated Neoplasms); 18: Infectious and Parasitic DDs (Systemic or unspecified sites); 19: Mental Diseases and Disorders; 20: Alcohol/Drug Use or Induced Mental Disorders; 21: Injuries, Poison And Toxic Effect of Drugs; 22: Burns; 23: Factors Influencing Health Status and Other Contacts with Health Services; 24: Multiple Significant Trauma; 25: Human Immunodeficiency Virus Infection) effects and 95% CIs of changes in morbidity and changes in the size of the end-of-life population on changes in hospital admissions

As a final robustness check, we estimated model (1) separately to explain the changes in hospital admissions in 23 MDCs based on the changes in the demand-side determinants in the entire population. Figure 5 illustrates the resultant changes in morbidity (left) and the changes in the end-of-life population (right) (comprehensive results are in Appendix Table 5). There was substantial variation among the MDCs. For instance, the end-of-life effect was three times larger than the average effect for “Poorly Differentiated Neoplasms” (MDC 17). Additionally, admissions for “Diseases and Disorders of the Respiratory System” (MDC 4) were largely determined by the population in their last year of life. In contrast, the changes in admissions for “Burns” (MDC 22) were not significantly associated with changes in the end-of-life population or with changes in morbidity. Moreover, admissions for “Multiple Significant Trauma” (MDC 24) had no significant association with changes in morbidity, since both MDCs are usually triggered by accidents. Conversely, admissions for “Mental Diseases and Disorders” (MDC 19) were strongly associated with changes in population morbidity but were not significantly associated with changes in the end-of-life population. Taken as a whole, these intuitive results further underscore that our estimations are robust.

### Predicted changes from 2008 to 2011

We use the estimated parameters from our model (Table 2, column 4) to predict the change in admissions. The change in the observable demand-side determinants (morbidity, surviving population and end-of-life population) predicted 21.5% of the change in admissions we observed from 2008 to 2011. Overall, reducing morbidity decreased admissions up to the age of 74 years. From 75 years and above, we predict increasing admissions due to worsened morbidity. Shifts in the surviving population decreased admissions in the younger age groups and strongly increased admissions, particularly due to there being more people in the age groups 70 years and above. The end-of-life effect was negligible up to an age of 60 years, but, in particular, from 85 years and above, it increased admissions because more people were in their last year of life.

## Discussion

With this study, we contribute to the literature by investigating the effects of changes in demand-side determinants on changes in hospital admissions in Germany. We used a market-wide, full-sample, longitudinal dataset that allows detailed observations of changes in the major demand-side determinants of the entire German population and the corresponding inpatient admissions for both publicly and privately insured patients. Germany is particularly interesting for such estimations given that it was one of the five OECD countries with the greatest percentage increase in hospital admissions and the largest rise in the absolute number of hospital admissions per capita for the period from 2008 to 2016 in spite of comparable life expectancies and population structures to other countries [29].

Due to the panel structure of our data, we controlled for unobserved heterogeneity and aimed for unbiased estimations. The “red herring” debate in this line of research has been based essentially on complementing age as a proxy for high health care utilization with a better proxy, which has been time to death. We, however, took a different approach and included health status, in terms of morbidity, in our estimations, as this is the variable that really matters. A unique feature of our study is its novel and very detailed measure of the entire population’s morbidity based on all outpatient claims for 80 important diseases. We pursued two approaches to overcome the endogeneity between changes in morbidity and changes in hospital admissions. First, we recalculated the RSA risk factor by restricting it to outpatient data and, second, we used instrumental variables. Another novelty of our study is its modelling of demographic changes by interacting age with shifts in the population structure for both the surviving and end-of-life populations.

We found that changes in morbidity had the largest impact on changes in hospital admissions. Changes in the size of the surviving population had the second largest impact, whereas changes in the size of the end-of-life population had the smallest. The dominant role of health status as a key driver of hospital admissions has also been confirmed by recent studies. When considered as a variable, health status turns out more important than time until death (i.e., the end-of-life effect) or ageing [30–33]. In studies that do not include health status, time until death presumably approximates high morbidity, especially among those with longer time until death. The end-of-life effect has little influence once it no longer acts as a proxy for morbidity [30], and indeed represents only the net proximity to death (separated from morbidity), which is mainly associated with hospitalizations that are undertaken to avert death [32]. We found that including the size of the population in their last year of life as a variable was sufficient to depict the end-of-life effect when controlling for morbidity. Changes in the size of the population in their second-to-last year of life had hardly any impact.

Our finding that, overall, the changes in the size of the end-of-life population had a smaller impact on hospital admissions than changes in the size of the surviving population seems plausible given the much larger number of survivors in our market-wide analysis (i.e., in the entire German population). In the period 2008–2011, we observed around 850,000 persons in their last year of life (predominantly in very old age groups) and 80,000,000 survivors, which led to 16,000,000 inpatient stays per year. The number of hospital stays exceeded the number of people in their last year of life by a factor of nearly 19.

We did not expect to observe substantial population ageing during the short period covered by our study. Nevertheless, our detailed observations of shifts in the population structure in over 400 counties helped us to assess the effect of ageing, since these were associated with manifold age-specific changes in hospital admissions. With its somewhat U-shaped association, the large impact for the age group of teenagers may reflect accident- or alcohol-related hospitalizations [34], which are not explained by morbidity. We found the largest increase in admissions when the 60–95 year age group increased in size—an observation that has also been confirmed by Baal and Wong [9]. As mentioned above, the effect for the groups 80 years and older was predominantly due to the increasing number of deceased. However, the large impact of changes in the size of the surviving population aged 60–69 years was probably due to a combination of two underlying reasons. First, the increase may reflect a retirement effect as the number of retired persons in this age group increased substantially. Caroli et al. [35] found that health care utilization increased after retirement partly due to decreasing opportunity costs. In Germany, every emergency department visit is associated with a 48.8% probability of being admitted to inpatient care. Thus, decreasing opportunity costs may be one reason for the strong increase seen in recent years in the number of (non-urgent) emergency department visits in Germany and the consequent rise in the number of inpatient admissions [36]. Second, the surviving population effect may also reflect medical innovations targeted at older age groups, which reinforce their demand for hospital admission [8]. Planned, elective admissions for people distant from death can potentially be found here, whereas the end-of-life effect depicts demand for severe, urgent admissions to avoid death.

Based on our empirical findings and the results of our robustness checks, we believe that our results are robust. The intuitive results for separate MDCs strengthen confidence in our estimations. The inclusion of personal preferences for risk aversion and the exclusion of children barely changed our results. We argue that the demand-side determinants included in our models capture the vast majority of change on the demand side. Taken together, however, the demand-side determinants explain only one-fifth of the growth in admissions we observed in Germany. In particular, decreases in morbidity were associated with a decrease in admissions.

Our results confirm the results of recent studies that have also found end-of-life and age to be significant predictors of hospital utilization [9, 14, 18, 19]. In line with van Baal and Wong [9], we found age-specific changes in utilization (e.g., increases in admissions are the greatest when the size of the population in the highest age groups increases). Nevertheless, the comparability of our findings to those of previous studies may be limited by differences in methods, setting or the exclusion of morbidity as a variable. However, several studies have included morbidity [18, 30, Hazra, Carreras, 30]. While Wouterse et al. [18] also found that higher morbidity was associated with higher hospital utilization, a major difference between their study and ours is that it observed morbidity only at the beginning of the period they analysed. As a result, they were unable to relate hospital utilization to changes in morbidity over time. Our study confirms the results of Hazra et al., Carreras et al. and Howdon and Rice—namely that morbidity is the most relevant demand-side determinant. Nevertheless, the effect sizes differ because Hazra et al. looked only at the elderly aged 80–95 years, Carreras et al. used a two-part model of the probability of use and positive expenditure in different groups of healthcare services, and Howdon and Rice did not measure the general morbidity of the population, but rather morbidity captured during hospital episodes.

Similar to Wong et al. [14], we estimated diagnostic category-specific models and also found heterogeneous and mostly significant end-of-life effects, with the strongest effect for cancers. While the level of disease captured by Wong et al. [14] was more precise, we were additionally able to estimate diagnostic category-specific morbidity effects. De Meijer et al. [19] concluded that HCE predictions based solely on changes in population characteristics explain only 21% of the growth actually observed, in line with our predicted 21.5%.

Our study has several important limitations. Our analysis is limited to inpatient care; although this is an important part of health care, different results may be found for other health care sectors. Also, the pattern of demand for hospital care in Germany may deviate from that in other health care systems. In addition, we would have preferred to have had a longer time series. Lastly, separate estimations of supply and demand might ignore important information in the data, such as growth in medical technology interacting with patient age.

## Conclusion

Research on the demand-side determinants of hospital utilization has undergone several stages of evolution. The first of these involved examining the impact of age, whereas the second found that proximity to death, rather than age, was important. Our study builds on these findings and, using an extensive market-wide dataset, provides evidence that morbidity is the main demand-side driver of hospital utilization. The results of our models can help policy makers set national or regional budgets for inpatient care. Due to the major role played by morbidity, policies aimed at other demand-side drivers, such as patient preferences, will be limited in their effect on cost containment. While morbidity could be reduced through improved outpatient care, for example for ambulatory case sensitive conditions, most demand-side factors are not easily influenced by policy [4]. Since our findings indicate that the vast majority of changes are not caused by demand-side determinants and presumably originate from the supply side, we conclude that policy makers in full coverage systems where hospital reimbursement is based on DRGs with the inherent incentive to increase (profitable) admissions should focus more on supply-side incentives. Policy makers in other health care systems should, however, be cautious when transferring these results. To generate findings more useful to other health systems, future studies may benefit from extending their analysis to the joint estimation of supply and demand in the inpatient market.

## Appendix

See Tables 4 and 5.

**Table 4:** First-stage results

Specification	FS	FS
Column	1	2
∆Unemployment	0.0292*** (22.40)	0.0289*** (22.21)
∆End-of-life population	0.000433*** (3.42)	− 0.00600* (− 2.08)
∆Surviving population	0.000663 (0.53)	
∆Surviving population in age group		
1–4		− 0.0164*** (− 5.43)
5–9		− 0.0004 (− 0.11)
10–14		0.0141** (3.39)
15–19		− 0.0162*** (− 5.13)
20–24		0.0087* (2.25)
25–29		− 0.0099* (− 2.03)
30–34		0.0062 (1.13)
35–39		0.0083 (1.58)
40–44		− 0.0149* (− 2.49)
45–49		0.0007 (0.10)
50–54		0.0018 (0.25)
55–59		0.0221** (3.31)
60–64		− 0.0034 (− 1.02)
65–69		0.0213*** (5.03)
70–74		− 0.0457*** (− 6.60)
75–79		0.0224** (3.35)
80–84		− 0.0097 (− 1.55)
85–89		0.0113* (2.30)
90–95		0.0009 (0.21)
∆End-of-life population in age group		
1–4		0.0008 (0.73)
5–9		0.003 (1.51)
10–14		− 0.0003 (− 0.19)
15–19		− 0.0002 (− 0.35)
20–24		− 0.0005 (− 0.84)
25–29		0.0017** (2.82)
30–34		− 0.0006 (− 1.10)
35–39		0.0003 (0.62)
40–44		0.0004 (1.42)
45–49		0.0004 (1.27)
50–54		0.0007 (1.91)
55–59		0.0013** (2.97)
60–64		0.0004 (0.65)
65–69		0.0003 (0.42)
70–74		− 0.0009 (− 0.79)
75–79		0.0003 (0.21)
80–84		− 0.002 (− 0.93)
85–89		0.0063* (2.59)
90–95		− 0.006* (-2.08)
${\alpha _i}$	Included	Included
${c_j}$	Included	Included
${\mu _t}=2009$	0.00656*** (28.90)	0.00660*** (28.90)
${\mu _t}=2010$	0.00397*** (18.43)	0.00404*** (18.69)
${\mu _t}=2011$	Base	Base
*F*-statistic	501.86	493.46
*N*	74,855	74,855

Numbers in brackets represent *t* values

Two-tailed significance levels: **p* < 0.05; ***p* < 0.01; ****p* < 0.001

**Table 5:** Determinants for MDCs

	∆End-of-life population	∆Morbidity	∆Surviving population
MDC 01	0.0242***	0.223***	0.138***
MDC 02	0.0041	0.133*	0.179***
MDC 03	0.0070**	0.141**	0.147***
MDC 04	0.0463***	0.117*	0.123***
MDC 05	0.0139***	0.0921*	0.153***
MDC 06	0.0171***	0.123***	0.182***
MDC 07	0.0345***	0.0887	0.0793**
MDC 08	0.0059***	0.086**	0.155***
MDC 09	0.0139***	0.0905	0.16***
MDC 10	0.0083*	0.188***	0.0946***
MDC 11	0.0145***	0.12*	0.164***
MDC 12	0.0046	0.0291	0.102***
MDC 13	0.0085***	0.0272	0.0627***
MDC 16	0.0162**	0.232***	0.0083
MDC 17	0.0508***	0.331***	0.0215
MDC 18	0.0336***	0.119	0.108***
MDC 19	0.0021	0.316***	0.0437
MDC 20	0.0073	− 0.025	0.0914**
MDC 21	0.0059	0.156*	0.17***
MDC 22	0.0020	− 0.024	− 0.000
MDC 23	0.0148**	0.229***	0.0499
MDC 24	0.0086*	− 0.012	0.0333
MDC 25	0.0061	− 0.000	0.0020

Two-tailed significance levels: **p* < 0.05; ***p* < 0.01; ****p* < 0.001

## Footnotes

1 Inpatient hospital stays have required a co-payment of €10 per day within the German SHI system since 2004.

2 We lose about 4% of observations matching postcode-level data in 2010-version counties.

3 For example, hospital admissions for 65-year-old men in the county of Cologne increased by one percent from 2008 to 2009.

4 For the population data, a log(x + 1) transformation accommodates the highly skewed distribution of these data.

5 For example, from 2008 to 2009, one percent more 65-year-old men in the county of Cologne were in their last year of life, and two percent more were not in their last year of life.

6 To obtain an identified equation, we follow the approach of Clark [Clark], who composed 5-year age groups. Additionally, five cohort effects with the smallest changes in admissions are assumed equal. We found robust results equalizing different or more cohort effects.
